# The Institutional Worker

**Published:** 1913-04-05

**Authors:** 


THE
### INSTITUTIONAL WORKER
**Being a Special Supplement to "The Hospital"**

### OUR BUREAU OF INFORMATION.

### Rules for Correspondents.

1. Every letter, on whatever subject, must be accompanied by the coupon to be cut from the back cover (inside page) of The Hospital, current issue, and must contain the name and address of the correspondent with pseudonym for publication if desired.
2. Replies by post cannot be given, save under exceptional circumstances at the Editor's discretion.
3. Letters from Approved Homes in reply to special needs published in the Bureau should state terms and full particulars, and be sent prepaid under cover to the Editor of the Bureau with name written across coupon for identification.
4. Proprietors of Homes which have not yet been entered on the List of Approved Homes, but have spare accommodation likely to suit special needs, are invited to write for an application form for registration. The fee for registration, which includes two announcements of the Home in the Bureau and other privileges, is 10s.
5. All communications to be addressed to the Editor of The Hospital, 28 Southampton Street, Strand, London, W.C., and marked "Bureau of Information."

### Invitation to Readers.

**The Editor is prepared to make known without charge the needs of any who may be sick or in difficulties, and to guide them in making choice of Homes for treatment or convalescence. Reduced terms can often be arranged with Homes on the Approved List for those unable to pay full fees. Queries are specially invited from those who are engaged in any kind of philanthropical work. A new edition of the leaflet describing the purposes of the Bureau of Information is now ready and will be sent free of charge on application.**

### Prospects of Male Nurses.

The post of dresser is always filled by medical students. You do not require any capital to be trained as male nurse, but the great difficulty is the scarcity of training schools, especially as you are just too old for the Royal Army Medical Corps. Write and try at the National Hospital, Queen Square, London, W.C., but there are few vacancies. The highest salary for a male nurse is about £4 4s. a week, but often only about £30 or £40 a year is offered for permanent posts, and employment is very precarious. We recommend you to take training in a mental hospital.—Eager.

### Church of England Nursing Association.

Write to the Institution for Nursing Sisters, 4 Devonshire Square, Bishopsgate, E.C. You do not say whether you are trained or whether you wish to be trained for work among the poor in connection with the Church, but in either case the Hon. Lady Superintendent may be able to receive you. The Bishop of London's address is St. James's Square, London, S.W.—W. S. Pentre.

### To Obtain a Post.

The only method is to advertise and to answer advertisements. Have you had your certificates and testimonials typed? If not, get a dozen or more copies done at once, and send a set, with a stamped envelope directed to yourself, for their return, whenever you apply for a post. It makes it much easier for employers to judge whether you are the kind of nurse they want. We fear salaries average rather low for these vacancies.—Nurse Rose.

### Training for Fever Nurse.

Write to the Hon. Secretary, Fever Nurses' Association, Plaistow Hospital, E. As you have had two years' general training as well as two years' fever training you are in a very good position as regards fever work, and we think you would find it more lucrative to stick to this.—Nurse Ethel B.

### Inspection of Hospitals.

There are no Commissioners who inspect hospitals. The Editor of The Hospital may consent, however, in the interests of managers and patients alike, to investigate at his own discretion well-authenticated reports of hospital mismanagement

### Salary of Matron.

In a convalescent home of the size you name, containing eighteen or twenty beds, and receiving both male and female patients, the matron should not be offered less than £50 a year, raising to £60 after a term of years during which she had proved her worth. In an establishment of this size fully as much administrative ability is required as in one containing twice the number of beds.—Chairman.

### Maternity Experience.

We fear there is no hospital or Home where you could be received and paid a salary with a view to gaining more experience before entering for the C.M.B. Examination. Even your previous maternity experience will not gain you admission to any such Home without paying a premium. At the Jessop Hospital for Women, Sheffield, and at the Maternity Hospital, Brownlow Hill, Liverpool, the fee is £15 15s. for the course. There is nothing less expensive.—Nurse C.

### Home Wanted.

The patient you describe is far better in an asylum, and you will be very ill-advised to attempt her removal. She requires night and day watching, with constant firmness and forbearance. In a private Home, unless a special attendant were engaged, she could not possibly be as well cared for as at present. The sum of such as the one to which you refer. For other query refer to Insurance columns.—John Bull.

### Matron in Boys' School.

These are of two kinds. Sometimes the post is merely that of housekeeper and needlewoman, to look after the boys' clothes, etc. In other cases it embraces the charge of the sanatorium, and is filled by a trained nurse. The former appointments are usually advertised in scholastic, the latter in nursing, papers.—Nurse C.

### Laundry Expenses n Sanatorium.

This is a Consumption Sanatorium of twenty-four beds, working-class patients only being taken.

In a selected month of four weeks the number of articles washed was 1920, at a cost of £4 5s. 4d., working out at ½d. plus two-fifths of a penny per unit.

Washing materials, wages of two maids, airing cost were 8d. per head per week, including staff.

As here the steam plant pumps the water supply, makes the electric light, and works the laundry, it is not possible to estimate the cost of power for the laundry only, or for the lighting; as the power exists for the other two purposes it may be estimated at nothing beyond the original outlay of laundry machinery. In my opinion, unless there existed a plant to do these things all at once, it would not be economical to put in steam machinery for laundry work only, though possibly more efficient. Where light and water are obtained from the main supply the cost of putting out the laundry is less.—Iota.

### Massage Training.

The difficulties you experience in arranging a scheme for the training of probationers in your infirmary likely to be accepted by the Board would be lessened if you could make an arrangement with a good outside school of massage to give the necessary instruction and lectures, allowing your probationers at the close of their regular training to take up this subject and make the necessary attendances. This plan has been tried in a first-rate London hospital with the greatest success. It is less expensive and far less troublesome than securing the necessary tuition inside the institution. We can procure you further particulars should you desire them.

It is not essential to extend the course of training to four years, but it is convenient to take the massage course after the conclusion of three years.—Matron R.

### A Nourishing Relish.

Have you ever tried pickled eggs as an expedient for combining piquancy with good food value? Take a wide jar and fill it with hard-boiled eggs, which should be as near new-laid as possible. Soak in boiling vinegar a little ginger, with allspice and a little garlic or such other flavouring as may be preferred. Fill the jar with the vinegar while still boiling, and cork it down for a few weeks. Serve as in the case of ordinary pickles, and you will find they will lend an interest to many dishes.—Sister G.

### Boarded-out Children.

Your proposal to receive boarded-out children needs very careful thinking out from a financial point of view. In the first place you must open up communication with a Board of Guardians desirous of boarding out a sufficient number of children for your purpose, and this can best be done through local interest. Approach the chairman or the board by letter in any district where you are well known and ascertain what terms they are prepared to give and the numbers they wish to arrange for. If you had one or two paying patients in addition you might manage it, but the patients who are willing to make part of a household for boarded-out children are not easily obtainable. Say you had twenty children at 8s. a week each, the total income would be £400 a year. But could you pay rent, taxes, coal, lighting, domestic expenses, laundry, wages and food for this big household on such a sum? We should be sorry to try. The food alone could hardly come to less than £300. On all grounds therefore caution is indicated.—Nurse B. H.

### To Utilise Rhubarb.

You will find that very few will reject this "vegetable" if cooked as follows: Stew gently in a modicum of water some young rhubarb, rub it through a sieve and flavour with sugar and lemon. Pour in a cup of hot water in which a sheet of gelatine has been melted. Line a mould with sponge cake, pour in the hot mixture, and leave to get cold. When turned out it can be covered with whipped cream or custard and garnished with ratafias. This makes a light and nourishing dish for convalescents.—Sara.

15s. which you are prepared to pay only covers the bare cost of her maintenance, leaving nothing over for service or rent of room. There are no charitable Homes where such cases are received. Mrs. S. T.

### Sanatorium for Reducing Weight.

The ordinary regime of diet and exercise prescribed both at English baths and the German ones you name has frequently a beneficial effect upon corpulency. But its radical cure is another matter. You will be wise to follow the advice of a doctor who knows the patient's constitution. The surgical treatment of obesity has been tried in America, and an article on the subject appeared last week in our columns.—G. P.

### Damages for Loss of Health.

There is no legal remedy, we fear, for the illness which you state is the direct outcome of insanitary office accommodation. The point was tried a short time ago, but without success. It accentuates the bad management that the authorities have undertaken the cost of a hundred-guinea operation to cure an official whose health might have been preserved if half this sum had been spent in alterations. We recommend you to make an impartial and courteous statement of the facts in the interests of the staff, and forward it to the chairman of the company with a request that he will personally investigate the circumstances and the condition of the office.—B. W.

### Training in Monthly Nursing.

At the British Lying-In Hospital, Endell Street, W.C., the course in monthly nursing lasts two months, and the fees are four and a half guineas for instruction and £4 a month for board and lodging. At the City of London Lying-In Hospital, City Road, E.C., the course lasts two months, and the fee is £12 12s. At the Clapham Maternity Hospital, Jeffrey's Road, Clapham, the course lasts three or four months, and the fees are from £11 11s. to £13 13s. At the General Lying-In Hospital, York Road, Lambeth, the course lasts three months, and the fee is £18 18s. At Queen Charlotte's Lying-in Hospital, Marylebone Road, N.W., the course lasts sixteen weeks, and the fee is £24. These are the principal schools, as you want to be trained in a hospital and in London.—C. F. C.

### Institution Facts and Figures.

### April Question.

Stating the number of beds in your institution and the class of patients received, give the following particulars :—

The number of garments washed during any four weeks in the winter months, with the price per unit, and the manner in which the cost was arrived at. In addition to wages, fuel, light and power, interest on capital should be taken into account. State the quantity, description, and cost of soap used in laundry for the month. If curd and oil soap be used, the amount of alkali should also be stated. State also the quantity of starch and blue.

The best answers will be published, and for each contribution considered by the Editor to be of sufficient interest for publication payment of Five Shillings will be made.

All letters to be addressed, with coupon enclosed, to The Editor, The Bureau of Information, 28-29 Southampton Street, Strand, London, W.C.

### Prejudice Against British Training.

We understand that British training is considered a drawback rather than a passport to favour in Mexico. This is the result of several bad failures which have occurred with English nurses at the Lady Cowdray Nursing Home, due to half-trained women posing as nurses, so it is earnestly to be hoped that you will prove your mettle if you succeed in obtaining employment in this town. The doctors are mostly German and American, and the latter naturally prefer American-trained nurses. Mexican nurses earn 10s. a day, and thoroughly trained foreign nurses as much as £1 a day. Write to the Superintendent of the Lady Cowdray Home, with copies of certificates and testimonials, and enclose coupon for reply. You must expect to find employment very precarious at first until you get known.—Nurse P.

### Good Nursing Prospects in Lisbon.

In times of pressure there are not sufficient trained nurses in Lisbon to meet all demands, but you cannot count on this as a regular condition. Terms range from two and a half guineas a week. The British Hospital employs two British nurses, and the Lisbon Nursing Association also has one salaried nurse. Conversational knowledge of Portuguese is a condition to success as a private nurse, but it is not a difficult language to master if you have a little French.—R. U. H.

### The Editor's Letter-Box.

### Communications have been received and attended to from:—

Miss R. G. C.; Nurse E. M.; Miss H. W.; Mrs. A.; Miss McC.; H. D.; Miss Mackie; Mrs. Bright; and others.

Sister Arnold, Invalids' Home, Milton Road, West Hartlepool.—We have received your letter and enclosure, and note you can receive two permanent patients at moderate terms. Glad to know your Home gains in comfortable adjuncts and that all goes well.

Successful Hospital Garden.—Yes, it is quite right, and you will hear in due course. Please let us hear from you again.

Miss E. B.—We will give you a legal opinion on your hard case shortly.

Nurse G. C. O.—We are unable to introduce to patients any but the Homes on our Approved List.

### APPROVED HOMES ADDED TO THE LIST.—January to March 1913.

**Berkshire.**— Rose Cottage, Cholsey, Wallingford. Principal: Sister Marion. Medical and slight mental cases. Aged people; delicate children. Comfortable Home in large garden at the foot of the Berkshire Downs.

**Bexhill-on-Sea.**—16 Parkhurst Road. Principal: Mrs. W. Hayman. Mental, epileptic, and chronic cases. Husband mental nurse and masseur. Home faces south, with sea view. Near station.

**Boscombe, Hants.**—Riffelberg, 10 Hamilton Road. Principal: Miss L. A. Fisher. Medical and surgical cases, rest-cure, chronic. Attractive central situation on high ground. Electrophone. Tel. 1169.

**Broadstairs.**— Two patients only received by experienced trained nurse, ladies or children. Special advantages for chronic invalids, deaf or blind patients. Pleasant sunny situation. Moderate terms.—DOMEN.

**Cornwall.**—Tregoddick, Flushing, Falmouth. Principal: Miss Kicke. For consumptive cases, who can be received in an advanced stage. Equable climate. Very sunny, cheerful situation, with view of harbour. Kindest care. Garden and shelter on edge of cliff.

**Harrogate.**—40 The Avenue, Starbeck. Principal: Miss Coulter. A very comfortable Home for medical, tubercular, chronic or slightly feeble-minded cases. Great experience and success with children. Healthy and bracing locality; garden.

**London, S.E.**—49 Catford Hill. Principal: Mrs. Ambler. Maternity, chronic, and rest-cure cases. Massage if required. Detached house, large and comfortable. Good garden. Open situation.

**London.**—198 Cromwell Road, South Kensington. Principal: Miss Bartholomew. Thoroughly high-class and fully-equipped Nursing Home, for medical, surgical, and other cases.

**London.**—131 and 133 Upper Richmond Road, Putney, S.W. Matron: Miss MacDonald. Fully-equipped Home for medical and surgical patients. Operating theatre. Chronic cases specially studied. Night nurses. Good gardens.

**London.**—56 Home Park Road, Wimbledon Park. Principal: Miss G. Morris. A private Home for medical, chronic, and rest-cure cases. Every comfort. Highly recommended by medical men. In healthy district.

**London.**— Ferndale, Cranfield Road, Brockley, S.E. Principals: Mr. and Mrs. Louis Lardent. Comfortable Home for patients needing massage, electrical and rest treatment. Moderate inclusive terms.

**Newbury.**—The Cottage, East Ilsley. Principal: Miss Goodley. Medical, surgical, slight mental, rest-cure cases, massage. Specially adapted for nervous, convalescent, and outdoor treatment. Nice garden.

**North Wales.**—Berwynfa, Llangollen. Principal: Miss Edward. Maternity cases, chronic and convalescent. Kindest care and home comforts. Sunny situation and fine air.

**Scotland.**— Albertville Nursing Home, Musselburgh. Principal: Mrs. Stirling. Medical, surgical, chronic, and convalescent cases. Good accommodation for elderly ladies or gentlemen. Garden; sea air; near good medical baths.

**Sussex.**— 57 Marina, St. Leonards-on-Sea. Principal: Miss South. Fully-equipped Medical and Surgical Home. Dowsing radiant heat and light treatment given. Good situation. Every comfort.

**Sussex.**—"Melrose," 77 St. Aubyns, Hove. Principal: Miss Thomas. Medical, maternity, rest-cure, and convalescent surgical cases. Private rooms for each patient. Large garden.

**Wimbledon.**—The Alexandra Homes, 153 Merton Road, and 4 Francis Grove, St. George's Road. Principal: Mrs. Perrin, with Miss Harriet Perrin. For chronic, senile, and bed-ridden patients in indigent circumstances, who can be received at from 10s. 6d. a week. Doctor attends Home. Garden.

### APPROVED HOMES DISCONTINUED.

Miss Richardson, Landholm, Totland Bay; Miss Mills, Mascot, Seaton, Devon; Sister Miriam, Brighton.

### Editor's Notices.

**Contributions:**

Contributions should be written, or preferably typed, on one side of the paper only, and all articles sent in are accepted upon the distinct understanding that they are forwarded to THE HOSPITAL only.

The Editor cannot undertake to return MSS. not used, but when a stamped directed envelope is enclosed the MSS. may be returned if a special request is made.

Accepted articles and paragraphs of news will be paid for after publication at the scale rate.

**Address:**

To prevent delay all contributions and letters on editorial business must be addressed exclusively to the Editor, "The Hospital," 29 Southampton Street, Strand, London, W.C. It is important that this regulation shall be strictly observed.

**Correspondence:**

Correspondence on all subjects is invited. The name and address of correspondents must be given as a guarantee of good faith, but not necessarily for publication.

**Special Articles:**

Special articles are invited, and questions, inquiries, and paragraphs upon all matters relating to the work, administration, and management of general, special, mental, fever, cottage and convalescent hospitals, sanatoria, homes, institutions, societies and organisations for the treatment or care of the sick, injured and dependants of all classes. Every matter affecting the interests and welfare of the staff of all grades working in these institutions will receive special consideration.

**Administrative Medicine, Research and Items of News:**

Special terms will be paid for approved articles on subjects in this wide field by experts who have made some section of it their special study and interest. We wish to give prominence to every movement tending to advance accuracy of diagnosis, efficiency in treatment, and the development of modern methods to eradicate disease and promote the welfare of the suffering.

**A Bureau of Information:**

We invite inquiries and applications for information and help in providing for that numerous class of sufferers who require special care or house-room which they cannot obtain in their own homes or secure for themselves. We cannot, however, prescribe, or recommend practitioners.

